# Governing the Global Commons with Local Institutions

**DOI:** 10.1371/journal.pone.0034051

**Published:** 2012-04-03

**Authors:** Todd Bodnar, Marcel Salathé

**Affiliations:** 1 Department of Computer Science and Engineering, Pennsylvania State University, University Park, Pennsylvania, United States of America; 2 Department of Biology, Pennsylvania State University, University Park, Pennsylvania, United States of America; University of Maribor, Slovenia

## Abstract

Most problems faced by modern human society have two characteristics in common - they are tragedy-of-the-commons type of problems, and they are global problems. Tragedy-of-the-commons type of problems are those where a commonly shared resource is overexploited by free riders at the expense of everyone sharing the resource. The exploitation of global resources such as clean air and water, political stability and peace, etc. underlies many of the most pressing human problems. Punishment of free riding behavior is one of the most frequently used strategies to combat the problem, but the spatial reach of sanctioning institutions is often more limited than the spatial effects of overexploitation. Here, we analyze a general game theoretical model to assess under what circumstances sanctioning institutions with limited reach can maintain the larger commons. We find that the effect of the spatial reach has a strong effect on whether and how the commons can be maintained, and that the transitions between those outcomes are characterized by phase transitions. The latter indicates that a small change in the reach of sanctioning systems can profoundly change the way the global commons can be managed.

## Introduction

Many of the most pressing problems of human society are global problems: environmental pollution, overpopulation, nuclear proliferation, global warming, etc. At their core, most of these problems present a tragedy-of-the-commons-like situation [1]–[5], where the maintenance of a common good requires an effort by everyone, but can easily be exploited by free-riders who benefit from the common good without paying for the effort. For example, clean natural resources in highly populated areas require some forms of waste control - the associated costs are generally shared by everyone in the area, but the benefits of the clean resources are also shared by everyone in the area. The problem arises when individuals start reaping the benefits of the resource without sharing the costs. Because such free-riding behavior is beneficial to the individual, the resource cannot be maintained in the long run, hence the tragedy of the commons [6]–[9].

In order to prevent the tragedy of the commons caused by free-riding behavior, a number of strategies have been proposed. In particular, punishment of free-riding has received considerable attention. In its simplest form, punishment emerges from individuals who are willing to punish exploiters at a personal cost to themselves. If the threat and cost of being punished are greater than the potential gain from exploiting, such a simple sanctioning system can temporarily maintain the commons [10]–[14]. However, because the act of punishment is costly to the punisher, punishment itself is susceptible to free-riding: individuals are best off if they can benefit from the punisher's efforts without having to pay the cost of punishment themselves. This so-called “second-order free rider problem” can in principle be addressed with the punishment of non-punishers, but this will ultimately lead to an “n^th^-order free rider problem” which cannot be solved by such a simple sanctioning system [15], [16] .

A more complex sanctioning system, where individuals contribute to a punishment pool, rather than paying the cost of punishment themselves, has shown to be a potential solution to this dilemma. In such a “pool punishment” system, exploiters are punished by an institution paid for by pool punishers [17], [18]. Notably, the cost of paying into this punishment pool must be paid even in the absence of exploiters (and thus in the absence of punishment). This is in stark contrast to the “peer punishment” system described above, where costs in the absence of exploiters are trivial. Another notable aspect about pool punishment is that it is easy to identify those who do not contribute to the punishment pool, and are thus second-order free riders. This allows pool punishment to escape the “n^th^-order free rider problem” and maintain the commons despite the apparent inefficiency caused by the continuous costs that accrue even in the absence of exploiters [19], [20]. Indeed, institutionalized punishment of exploiters has been implemented on various levels of societal organization, ranging from so-called committees of vigilance in the American Old West to modern international criminal tribunals [21]–[23].

As the commons of the 21st century have become increasingly global (e.g. global climate change, global health, international terrorism), a problem of scale has emerged [24]. While exploitation of the commons now has far-reaching consequences across international borders, the reach of institutions sanctioning exploiters has not always kept pace. For example, local emission of greenhouse gases can have far-reaching consequences for the global climate, but international consensus on emission standards - and on the sanctioning of violations thereof - has been largely lacking [25]. At present, how the problem of scale affects the ability of sanctioning systems to govern the commons is not well understood. Building on earlier work using spatial evolutionary game theory, [26]–[28] we show that the type of sanctioning system that can be stably maintained depends strongly on the reach of the sanctioning system relative to the reach of the commons. Most importantly, we observe phase transitions between the dominant strategies such that small changes in the reach of the sanctioning systems can have profound impacts on how the commons are (or are not) protected against exploitation.

## Results

To investigate the effect of sanctioning systems with limited reach, we consider a spatial environment with a total of *M* players on a square lattice with *M* cells (implemented as a torus), where each player occupies a cell. We assume that the players do not move between games. Players can participate in a game if they are no more than an interaction distance *p_i_* away from a focal player. To play a game, players can contribute and receive from the commons as described above. Punishers can only punish other participants that are no more than a punishment distance *p_p_* away from the punishing player. Since the game is non-compulsory, players are given the option to stay out of the game and instead receive a payoff *σ*. The probability that two randomly chosen players of a game are able to punish each other is parameterized as a coverage coefficient *C_c_*, which can be calculated as
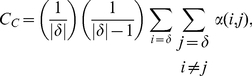
(1)where *δ* is a set of players within the interaction range of the initiating player, and *α(i,j)* is one if and only if the distance between players *i* and *j* is less than or equal to *p_p_* and zero otherwise. From this we can calculate the effective values of *b*, *β*, *g* and *γ* such that *b′ = b C_C_*, *β′ = β*, *g′ = g C_C_*, and *γ′ = γ C_C_.* Note that the cost paid by pool punishers is unmodified because they contribute to the punishing pool even when there are no defectors within their punishment range.

**Figure 1:**
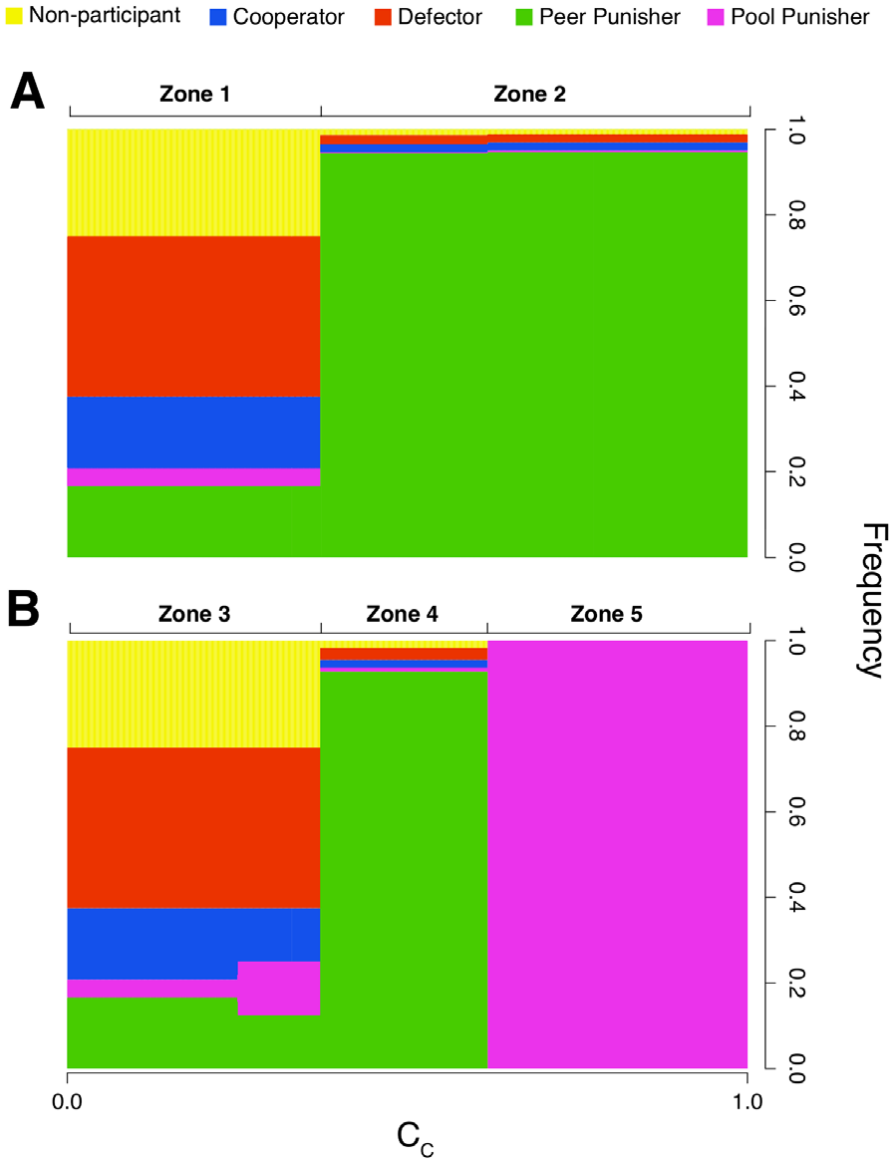
The Effects of *C_C_* on Strategy Frequency. Results of the mathematical model show that there are phase transitions between different zones of strategy distributions when *C_C_* is varied in games with only first order punishment *(* ***A*** *)* and in games with second order punishment *(* ***B*** *)*. Punishment is ineffective in zones 1 and 3. Peer punishment is effective in zones 2 and 4, and pool punishment is only effective in zone 5.

**Figure 2:**
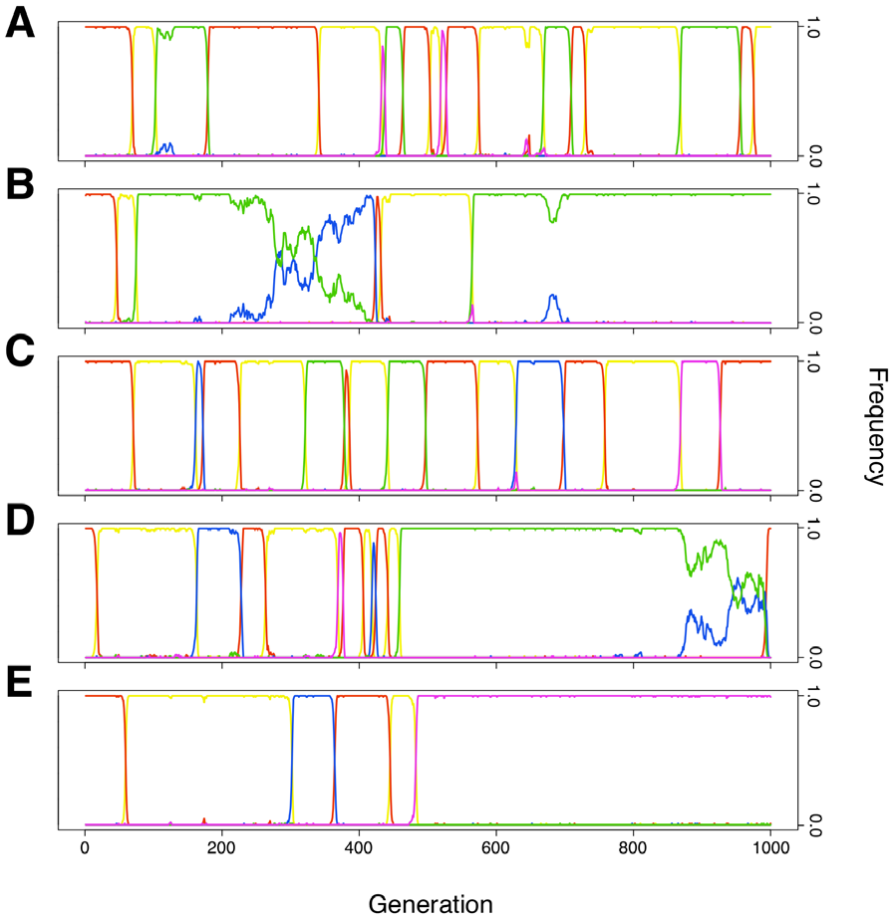
Examples of the Population Dynamics. Computational simulations of populations with parameters representative of zones 1 through 5 in Figure 1. In *(* ***A*** *)* and *(* ***C*** *)*, the reach of the sanctioning systems is not far enough to have any effect, and the population can cycle through all of the strategies. In panel *(* ***B*** *)* and *(* ***D*** *)*, peer punishment is able to stop an invasion of defectors, but does not stop non-punishing cooperation from drifting in. Once there are sufficiently few peer punishers, defectors can invade. Second order pool punishment is able to stop the invasion of non-punishing cooperation, hence pool punishment becomes locked in panel *(* ***E*** *)*. Color code as in Figure 1.

From this, we can now determine under what circumstances the commons can be maintained by punishment (without punishers, cooperators would always be invaded by defectors). In a non-compulsory game, defectors will always be invaded by non-participants [29]. In the case of first-order punishment, both peer punishers and pool punishers can invade non-participants. However, since we assume *β*>0, pool punishers have a lower payoff than the other cooperating strategies and thus can be invaded by cooperators or peer punishers. Then, defectors can invade a population of peer punishers if
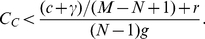
(2)Under this condition, the benefit from not contributing to the commons is greater than the cost of being punished. Thus, every strategy can be invaded by another strategy, and cooperation cannot be maintained (Figure 1A, zone 1, and Figure 2A). If the condition is not met, however, the commons can be maintained by peer punishment (Figure 1A, zone 2, and Figure 2B).

In the case of second-order punishment, peer punishment is ineffective as long as (2) holds. Pool punishers have to pay *β* even if they are not currently punishing any players, and thus they can be invaded as long as
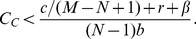
(3)In this case, pool punishers are unable to maintain the commons (Figure 1B, zone 3 and 4, and Figure 2C). If the condition is not met, however, the commons can be maintained by pool punishment (Figure 1B, zone 5, and Figure 2E). It then follows that peer punishers can maintain the commons for intermediate values of *C_C_* when
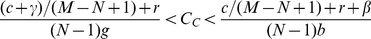
(4)If we assume that punishment costs are identical in both punishment systems (i.e. *g = b*), the range of *C_C_* for which (4) can be met will be non-zero (Figure 1B, zone 4, and Figure 2D). Thus, under some circumstances, peer punishment can maintain the commons even if second order punishment by pool punishers is permitted.

## Discussion

We've presented here a simple model of a spatial public goods game to address the problem of scale that has emerged as many of the most pressing issues facing modern societies have become global. While the simplicity of the model cannot capture every detail of the real world complexities inherent to governing the commons, it allows us to formalize the effects of some key parameters that are common to all sanctioning institutions, such as the costs associated with punishment. In particular, as can be seen from Eq. (4), in order for pool punishment to emerge under even small values of *C_C_*, *β* should be small whereas *b* should be large. That is, in order to protect the global commons with non-global sanctioning systems, the contribution cost to a sanctioning institution should be minimized while the costs imposed on non-cooperators should be maximized [9]. Thus, the model can replicate our intuition about the incentive structure of sanctioning systems. However, through the addition of spatial constraints, unexpected threshold phenomena emerge such that small changes in the system can lead to the fundamental changes. For example, inequalities (2)–(4) demonstrate how small changes in *C_C_* can have profound effects on whether the commons can be maintained, and if so, by which type of sanctioning system. Furthermore, while previous work has argued that peer punishment is outcompeted by pool punishment when second order punishment is permitted [19], our results indicate that peer punishment wins when the reach of the sanctioning system is intermediate relative to the size of the commons, a situation that is likely to be relevant in many applications. Future work may extend this model to include the effect of rewards on cooperation [30].

**Figure 3:**
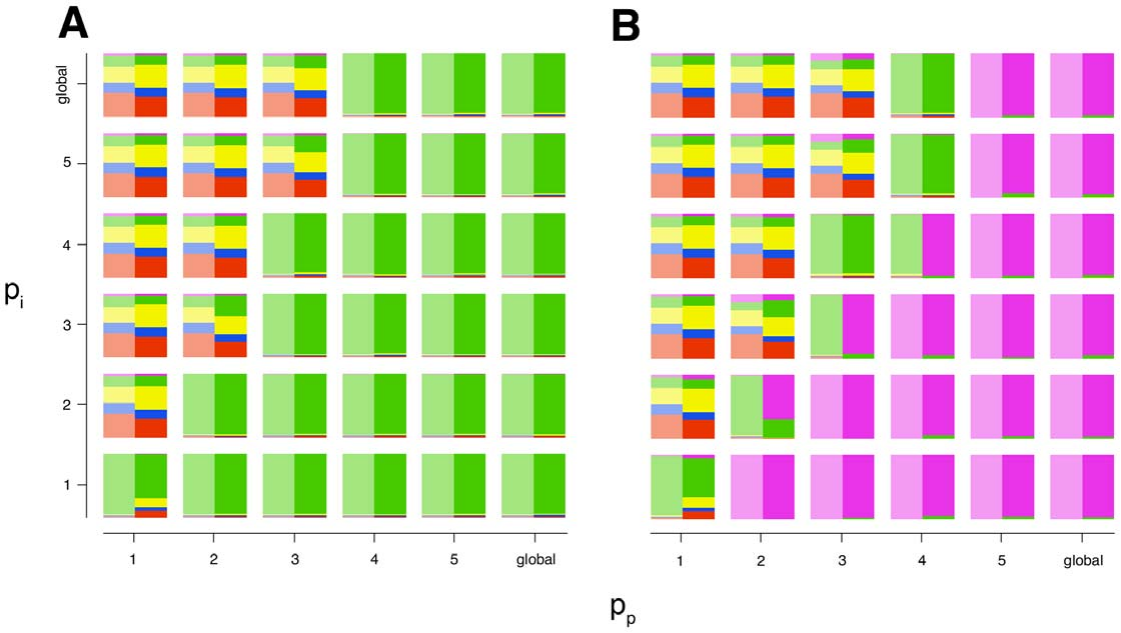
Strategy Frequencies for Non-Global Games. Results from the mathematical model (semi-transparent) and computational simulations (non-transparent) for non-global games with only first order punishment *(* ***A*** *)* and with second order punishment allowed *(* ***B*** *)*. Note the similar phase transitions in non-global games. Color code as in Figure 1.

As we've shown above, in order to protect the global commons from exploitation, the reach of sanctioning institutions must be sufficiently long. This observation also holds for commons that are limited in size (i.e. where *p_i_*<∞) (Figure 3). Overall, the commons always benefits from sanctioning institutions with long reach.

## Materials and Methods

We developed a simple model implementing a spatial public goods game that reflects the fundamental dilemma of the tragedy of the commons. A public goods game models individuals' contributions to the common good. Specifically, each player is given the choice to donate to the common good or not. These contributions are then multiplied by a constant and evenly divided amongst the other players. Because a player receives the same amount regardless of its own donation, a rational player would not contribute to the commons. More formally: in each game, an initial focal player is selected randomly. Then a set of *N-1* other players that are no more than an interaction distance *p_i_* away from the focal player partake in a game with the focal player. In this model, a player cooperates by donating an amount *c* to a commons. This donation is then multiplied in value by a factor *r* and evenly distributed to the rest of the participants [7]. A defecting (non-cooperating) player does not contribute to the commons, but still receives the same benefit as all the other players. Thus, in a compulsory game, without any form of punishment, cooperation would quickly deteriorate. In a non-compulsory game, a rock-paper-scissors–like cycle of non-participants, cooperators, and defectors emerges, where phases of cooperation are short-lived [29], [31], [32]. We allow punishment to take two forms, peer or pool punishment [19]. Peer punishers devote resources to directly punish defectors themselves [33], whereas pool punishers devote resources to a punishment pool that pays for institutionalized punishment upfront [17].

### Playing the Game

We begin by defining a very basic public good game where all of the *N* players cooperate by contributing *c* which is then multiplied by a constant *r* and equally divided among the other players. In this case, all players will receive a payoff of
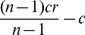
(5)We allow for the possibility of players not contributing to the pool, and thus being defectors. Each defector receives the same amount from the resource pool as a cooperator does, but without paying to donate to the pool. Thus in a game with *X*

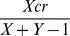
(6)cooperators and *Y* defectors, a defector will receive a payoff of , and a cooperator will receive a payoff of ,
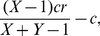
(7)Furthermore, we allow for a player to not participate in the game at all and to receive a payoff of *σ* instead of the part of the resource pool that a player would otherwise receive [6]. We will assume that a participant will be forced to be a non-participant if there are no other participants within the group that it is attempting to play with. Given a world with *X* cooperators, *Y* defectors, and *Z* nonparticipants, the probability that a participant will not be able to play with any other players is
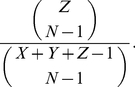
(8)Note that if *X*+*Y*>1, there is a non-zero probability of a game taking place, and it is assumed that a game will take place given a sufficient number of attempted games.

### Peer Punishment

We will now allow for the possibility of peer punishment. A peer punisher is a cooperator that fines every defector that it plays with a penalty *g*. To engage in this punishment, a peer punisher must pay *γ* per defector punished. In a game with *X* cooperators, *Y* defectors, and *W* peer punishers, a cooperator will receive a payoff of
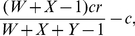
(9)a defector will receive a payoff of
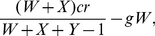
(10)and a peer punisher will receive a payoff of
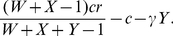
(11)It is in principle possible that peer punishers would punish cooperators, because regular cooperators do not contribute to the second order common good of punishing defectors. However, we assumed that at most two strategies exist at any time. In a world of non-punishing cooperators and peer punishers, there will not be any defectors, so the behavior of a non-punishing cooperator will be the same as a peer punisher. Thus, peer punishers would be unable to differentiate between other peer punishers and non-punishing cooperators making second order peer punishment impracticable [19].

### Pool Punishment

We can also allow for pool punishment. A pool punisher contributes *β* each round to maintain a punishment pool. This punishment pool then fines every defector by amount *b* for every pool punisher the defector cheats. If a game is played with *V* pool punishers, *W* peer punishers, *X* cooperators, and *Y* defectors, a pool punisher will receive a payoff of
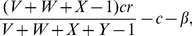
(12)a peer punisher will receive a payoff of
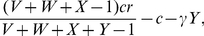
(13)a cooperator will receive a payoff of
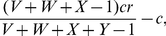
(14)and a defector will receive a payoff of
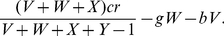
(15)Unlike peer punishment, there is a noticeable difference between pool punishers and other cooperators even in the absence of defectors. This allows for pool punishers to engage in second order punishment. We can account for second order punishment by simply subtracting *bV* from (13) and (14). Since peer punishers can differentiate between pool punishers and themselves, it is possible that peer punishers can engage in second order punishment of pool punishers also. However, this does not effect the dynamics.

### Population Dynamics

Consider two players *i* and *j* with payoffs *P_i_* and *P_j_*. We can use a simple social learning function
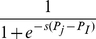
(16)to define the probability that player *i* will imitate player *j*. We will assume that each player will randomly choose another player to consider updating. Note that if *s→∞*, player *i* will always imitate player *j* when *P_j_>P_i_*, and player *i* will imitate player *j* with a 50% probability when *P_j_ = P_i_*. Otherwise, player *i* will not imitate player *j*. Furthermore, a player may randomly switch to a new strategy with a probability *μ*. We will assume that *μ* is small enough that mutation is rare and likely to occur only when the population's strategies are uniform [34], [35].

Now consider a world with *M* players where *M – 1* players have the same strategy, and one player has a new strategy. As before, the players divide into groups of *N* players to play a game. After these games have been played, a player is chosen to update using (16). We can model the frequency of the new strategy using a birth and death process [36]. Clearly, the states where there are either zero or *M* players with the new strategy are absorbing states. The probability that the new strategy successfully invades the population is the probability that the random walk reaches the latter state. We define this probability as *P_i→j_*.

For a new strategy to invade a world with *M* players that have the same strategy, one of the players must first mutate into the new strategy, then that strategy must replace the other *M–1* players' strategies as shown above. In our model, there are a total of 5 strategies, so the probability that a mutation will result in a specific strategy is 1/4. From this, we can build a 5 state Markov chain where each state represents a uniform
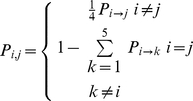
(17)population of a specific strategy, and the transition probabilities are defined as

We can then take the limiting distribution of the chain to find the long term average frequency of each of the strategies [20].

### The Coverage Coefficient

Given a location and a play distance, we can find a set *δ* of players in that area. The N players that will play together are randomly chosen from this set. However, some of these players may not be within the punishment distance *P_P_* of each other, so their ability to punish will be lowered. The probability *C_C_* that two players randomly chosen from the set are able to punish each other is
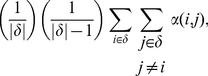
(18)Where *α(i, j)* is one if the euclidian distance between players *i* and *j* is less than *P_P_* and zero otherwise. Since the two players are not always able to punish each other, their effective values of *b*, *β*, *g* and *γ* are
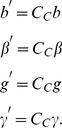
(19)Note that the amount pool punishers pay to maintain the punishment pool is unchanged because they must donate every round even if they do not punish any players. Thus pool punishment becomes less efficient as *C_C_* decreases.

### Phase Transitions

Regardless of whether second order punishment is allowed, we see a sharp transition from a fairly even spread of strategies to peer punishers being the dominant strategy. This transition occurs because peer punishment is unable to prevent the invasion of defectors when *C_C_* is below the a certain point. Let us begin with a single defector trying to invade a population of peer punishers. If we assume that *M* is much larger than *N*, it is unlikely that the defector will try to imitate one of the players it cheated, but instead a peer punisher who's payoff was not lowered because of being cheated or having to pay to punish. The cheated peer punishers are also more likely to consider imitating someone other than the defector. If there is more than one defector, the probability of imitating a player that is not a defector or has not been cheated goes down, and defectors have an easier time spreading. Thus, the ability of peer punishers to sufficiently punish the initial defector determines which side of the phase transition the game is on. By combining (10) and (11) and using the effective parameters defined in (19), we can show that this transition occurs at
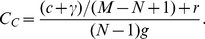
(20)When second order punishment is permitted, another phase transition is observed. This transition is at a higher level of *C_C_* and separates the values of *C_C_* where pool punishers are able to stop defectors from invading and where they are not. The reason that this transition happens at a higher value of *C_C_* than the one for peer punishers is because all pool punishers contribute to punishment, whereas only peer punishers that are actively punishing have their payoffs lowered. As with (20), we can use (12), (15) and (19) to show that this transition occurs at
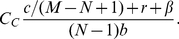
(21)We observe that peer punishers dominate the second order game for medium values of *C_C_*.
